# The Emerging Role of Cyclin-Dependent Kinases (CDKs) in Pancreatic Ductal Adenocarcinoma

**DOI:** 10.3390/ijms19103219

**Published:** 2018-10-18

**Authors:** Balbina García-Reyes, Anna-Laura Kretz, Jan-Philipp Ruff, Silvia von Karstedt, Andreas Hillenbrand, Uwe Knippschild, Doris Henne-Bruns, Johannes Lemke

**Affiliations:** 1 Department of General and Visceral Surgery, Ulm University Hospital, Albert-Einstein-Allee 23, 89081 Ulm, Germany; balgarciareyes@gmail.com (B.G.-R.); annalaura.kretz@googlemail.com (A.-L.K.); jan-philipp.ruff@gmx.de (J.-P.R.); andreas.hillenbrand@uniklinik-ulm.de (A.H.); uwe.knippschild@uniklinik-ulm.de (U.K.); doris.henne-bruns@uniklinik-ulm.de (D.H.-B.); 2 Department of Translational Genomics, University Hospital Cologne, Weyertal 115b, 50931 Cologne, Germany; s.vonkarstedt@uni-koeln.de; 3 Cologne Excellence Cluster on Cellular Stress Response in Aging-Associated Diseases (CECAD), University of Cologne, Joseph-Stelzmann-Straße 26, 50931 Cologne, Germany

**Keywords:** pancreatic cancer, pancreatic ductal adenocarcinoma, CDK, cyclin-dependent kinase

## Abstract

The family of cyclin-dependent kinases (CDKs) has critical functions in cell cycle regulation and controlling of transcriptional elongation. Moreover, dysregulated CDKs have been linked to cancer initiation and progression. Pharmacological CDK inhibition has recently emerged as a novel and promising approach in cancer therapy. This idea is of particular interest to combat pancreatic ductal adenocarcinoma (PDAC), a cancer entity with a dismal prognosis which is owed mainly to PDAC’s resistance to conventional therapies. Here, we review the current knowledge of CDK biology, its role in cancer and the therapeutic potential to target CDKs as a novel treatment strategy for PDAC.

## 1. Background

Despite advances in diagnosis and therapy, carcinoma of the exocrine pancreas, especially pancreatic ductal adenocarcinoma (PDAC), imposes a grand challenge for oncology. PDAC is one out of only two cancers where there has been virtually no progress in survival rates over the last decades, the second one being lung cancer [1]. Because pancreatic cancer presents with non-specific symptoms, like diffuse abdominal discomfort and loss of appetite [2], it is often only diagnosed at a late stage, when it is more difficult to treat [3].

With an incidence of 4.2 per 100,000, pancreatic cancer is the 12th-most common cancer in humans [4]. Within malignancies of the gastrointestinal tract, PDAC ranks second behind the most commonly occurring colorectal cancer [1,5]. PDAC is one of the deadliest cancer entities, with a cumulative five-year-survival rate of only 8% [1,3]. This meagre survival is partly caused by late diagnosis as suggested by the fact that a solely localised tumour without metastases offers a four-times improved survival rate of 28% [1]. In cancer-associated mortality statistics, pancreatic cancer ranks fourth in both sexes [1]. It is a cancer entity associated with old age, as pancreatic cancer arises most frequently in the seventh and eighth decade, although, less frequently, it is also reported in people under 40 years of age [6].

The only potential cure for PDAC is surgical resection [2,7,8]. However, this possibility is hampered by the aggressiveness of this disease, presenting early spreading, invasion, distant metastases, and a typical resistance towards conventional radio- and chemotherapy [2,3,9,10,11]. At the time of diagnosis, only 15 to 20% of pancreatic cancers are operable (i.e., have not spread to other organs), whereas the rest can only be treated through palliative chemotherapy, often with unfavourable results [12]. Most patients that have been treated with curative intent develop on-site relapse and distant metastases [2,13]. Therefore, clinicians are in need of adequate and effective chemotherapeutic regimens. Adjuvant treatments are mostly based on gemcitabine; palliative disease is mostly treated with gemcitabine or FOLFIRINOX (FOL = Folinic acid = Leucovorin, F = 5-FU = 5-Fluorouracil, IRIN = Irinotecan, OX = Oxaliplatin) or tyrosine-kinase inhibitor erlotinib [14,15].

The prodrug gemcitabine has been the gold standard for adjuvant treatment for a long time. However, the effects on improving median survival are only marginal at best [16,17]. Paclitaxel, nowadays prepared as nab-paclitaxel (nanoparticle albumin-bound), combined with gemcitabine improved therapeutic success, although not to a satisfactory level [18,19,20]. FOLFIRINOX has been a second line regime for advanced and metastatic pancreatic cancer since its introduction as PDAC treatment a few years ago [21,22]. However, new trends [23] indicate FOLFIRINOX as a first-line medicament in curative and adjuvant settings, as it was shown to be superior to gemcitabine, as it had a significantly better overall survival, disease-free survival, and metastasis-free survival during a randomized clinical trial [24,25].

Unfortunately, gemcitabine, nab-paclitaxel, and FOLFIRINOX are highly toxic [13,20,26,27]. In addition, currently approved therapies for PDAC yield unsatisfactory results. Hence, new therapeutic strategies with lowered toxicity and higher on-target efficacy are needed. Cyclin-dependent kinases (CDKs) have been proposed as possible targets of pharmacological inhibition for several cancer entities. Indeed, there are some successful examples of CDK inhibition in clinical settings, particularly against breast cancer, non-small-cell lung cancer, melanoma, and head and neck squamous cell carcinoma (reviewed in [28]). In the case of pancreatic cancer, the available clinical and pre-clinical evidence for a benefit in inhibiting CDKs is still scarce, although CDKs play an important role in the pathobiology of this disease.

## 2. Family of CDKs

Cyclin-dependent kinases (CDKs) are serine/threonine kinases that control cell cycle progression and other critical functions within the cell. Based on the homology of their catalytic domains, CDKs, along with the Mitogen-activated protein kinases (MAPKs), Glycogen synthase kinase-3 β (Gsk3β), members of the dual-specificity tyrosine-regulated kinase (DYRK) family and CDK-like kinases belong to the CMGC group of kinases (named after the initials of its members) [29,30]. CDKs are activated by cyclins and act as a regulatory subunit. These CDK/cyclin complexes are fundamental to the orderly progression of the cell [31]. Studies examining the functions of CDKs and cyclins have uncovered that in fact, these proteins have other relevant roles besides regulation of the cell cycle [31,32]. These other roles include, for example, regulation of transcription, epigenetic regulation, metabolism, stem cell renewal, and spermatogenesis [33,34,35,36].

According to the classification of Malumbres et al. [37], there are 21 CDKs (see Table 1), sharing a conserved catalytic domain that comprises an ATP-binding pocket, the amino acid sequence PSTAIRE as a cyclin-binding domain and an activating T-loop motif. Active CDK/cyclin complexes depend on the phosphorylation of the T-loop on the respective CDK. Cyclins interact with the PSTAIRE helix, displacing the T-loop and exposing the substrate binding domain of the kinase thereby allowing for its phosphorylation. Unlike CDKs, cyclins are heterogeneous proteins, characterised by the presence of the so-called cyclin-box, which mediates binding to CDKs [38]. Outside this domain, their sequences are quite diverse. Most known cyclins promote CDK activity. There are several regulatory mechanisms for CDKs at the post-transcriptional level, CDK inhibitors exerting a negative regulation, phosphorylation status, protein folding, and subcellular localisation [39,40,41]. Phosphorylation can regulate CDKs in a negative and positive manner [42]. For example, CDK1 has inhibitory (threonine 14, T14; tyrosine 15, Y15 in CDK1) and stimulatory (threonine 161, T161 in CDK1) phosphorylation sites [43,44]. Phosphorylation at T14 and Y15 within the ATP-binding site by inhibitory kinases Wee1 and Myt1 interferes with proper ATP alignment, whereas T-loop phosphorylation at T161 by CDK-activating kinases (CAKs) improves substrate binding and complex stability to enable full CDK activation [42]. In this review, a summarized overview of the role of CDKs during cell cycle and transcription will be provided.

## 3. Cell Cycle Regulation by CDKs

**Figure 1:**
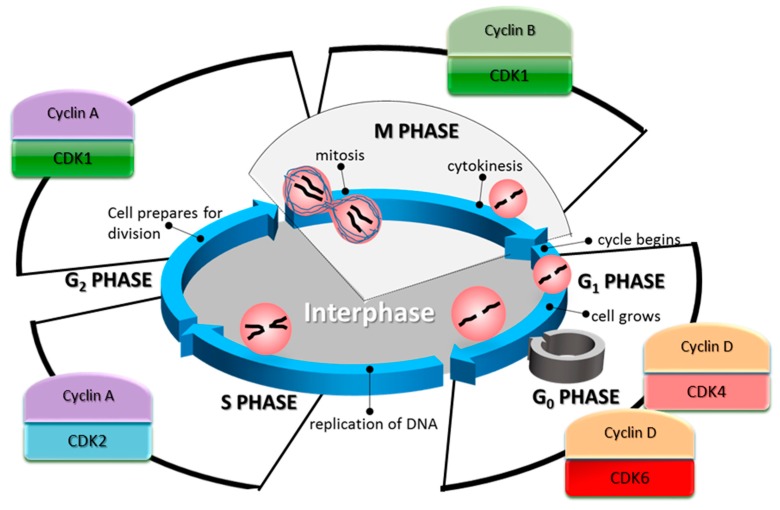
The cell cycle phases and their associated cyclin-dependent kinases (CDK)/cyclin complexes. In the G1 phase of the cell cycle, the synthesis of cyclin D is increased. This cyclin partners with CDK4/6 to promote cell cycle entry, and its progression through G1, as well as the G1/S transition. During the S phase, CDK2 in complex with cyclin A controls the phosphorylation of targets involved in DNA replication. Cyclin A is found highly expressed in this phase and until the last stages of G2. In the G2 phase, the primary regulator of the cell cycle is CDK1.

The cell cycle is one of the most essential and evolutionarily conserved cellular processes. CDKs and cyclins are the main proteins responsible for driving it [93,94,95]. Typically, these protein families act as heterodimers regulating different steps of the cell cycle, as CDK/cyclin complexes (Figure 1). CDKs unbound to cyclins are usually inactive, but upon the formation of a complex with a cyclin partner, they become able to phosphorylate different targets, resulting in target inactivation or repression. CDKs are constitutively expressed in the cell while cyclin expression is highly dependent on the stage of the cell cycle, hence their name. This fluctuating expression pattern of cyclins constitutes a mechanism by which the orderly progression of the cell cycle is regulated.

In the G1 phase of the cell cycle, the synthesis of cyclin D is stimulated. This cyclin is a partner of CDKs 4 and 6 [96]. Complexes of these CDKs and cyclin D promote cell cycle entry, progression through G1, and G1/S transition [56,97]. Cyclin D regulation is complex, depending mainly on the RAS/RAF/MEK/ERK signalling pathway for its transcription [98,99]. It is also regulated by its constant rate of degradation through the proteasome, mediated by GSK3β [100]. GSK3β phosphorylates cyclin D1, which primes it for its subsequent ubiquitination. The primary cell cycle-related target of the CDK4/6-cyclin D complex is the Retinoblastoma protein (Rb). Rb is one of the main repressors of the G1 to S phase transition [101], through its inhibitory activity on E2F, a transcription factor that promotes the expression of genes required for DNA synthesis and mitosis [102]. In humans, Rb contains 16 known phosphorylation sites that are targeted by CDKs [103,104], and its phosphorylation status varies along the cell cycle [105]. In its hypophosphorylated form, Rb can form a complex with E2F and repress its activity. Besides CDK4/cyclin D and CDK6/cyclin D, two other related complexes are known for their ability to hyperphosphorylate Rb: CDK2/cyclin E during G1, and CDK2/cyclin A during S phase [106].

One of the transcriptional targets of E2F is *CCNE1* encoding cyclin E [107], which in turn represses Rb when is in a heterodimer with CDK2 [56]. CDK2/Cyclin E complexes act mainly in the G1 and S phases of the cell cycle [96], however, its levels are reduced after the early stages of the S phase by proteasomal degradation. This particular complex has a broader range of targets related to the cell cycle, such as DNA replication and centrosome duplication [108]. CDK2 in complex with cyclins A1 or A2 is associated with S phase, with A2 being ubiquitously expressed in mice germ cells [109]. During this phase, cyclin A partners with CDK2 to phosphorylate targets involved in DNA replication [110]. Cyclin A is found highly expressed in this phase and until the last stages of G2. At the G1/S checkpoint, the cell halts its progression in the cell cycle if the conditions are not favourable for division. This checkpoint is partly controlled by the inhibition of the CDK4/cyclin D complex by the Inhibitor of CDK4 (INK4) family. These inhibitors competitively bind to CDK4 and CDK6, preventing, in turn, their binding to cyclin D, which is then degraded [100]. Either growth-induced or oncogene-induced overexpression of cyclin D alters this dynamic and pushes the cell towards the S phase [111].

In G2 phase, after the cell has duplicated its DNA during S phase, the primary regulator of the cell cycle is the complex formed between CDK1 and cyclin B. So far, more than 70 proteins have been identified as cellular targets of phosphorylation mediated by this complex [94], influencing many cell cycle-critical events, such as the separation of centrosomes [112], the condensation of chromosomes [113], breakdown of the nuclear lamina [114], and disassembly of the Golgi apparatus [115]. The activation of the CDK1/cyclin B complex is inhibited when DNA damage of genotoxic stress is present [116]. Also, its subcellular localisation is a regulation mechanism. CDK1 can be sequestered in the cytoplasm by the protein 14-3-3σ when it is separated from its partner cyclin B, either by competitive binding with p21Cip1 or directly dissociated by the Growth Arrest and DNA Damage-inducible GADD45 [117].

This complex network of CDK/cyclin interactions is not fully understood, not only because many other functions of these proteins have emerged in recent years, but also because there are many instances of functional redundancy in the cell cycle. For example, in the absence of CDK4/6, CDK2 can take over their functions when in complex with cyclin D [36]. In a similar manner, CDK1 can substitute for CDK2 and 4. In fact, the only essential CDK in the cell cycle is CDK1 which cannot be substituted for by another CDK [48]. In the absence of CDK2, CDK3, CDK4, and CDK6 in mouse embryos, CDK1 was able to bind to all cyclins, leading to the phosphorylation of Rb, an event required for cell cycle progression. However, the embryos were unable to develop past the morula and blastocyst stages in the absence of CDK1, showing that this CDK can drive cell division by itself [48].

## 4. Transcriptional Regulation by CDKs

**Figure 2:**
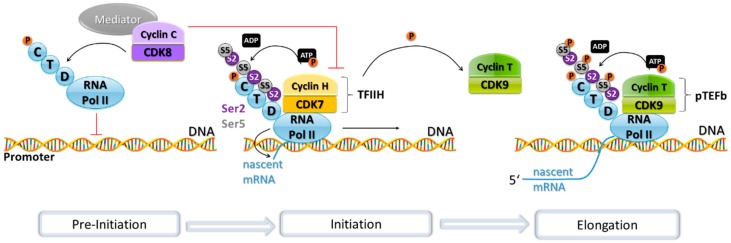
Transcription and its associated CDK/cyclin complexes. RNA Pol II forms part of the pre-initiation complex that starts gene transcription in eukaryotes. This pre-initiation complex is inhibited by the Mediator complex, of which CDK8 and cyclin C are part. The Mediator complex represses transcription by phosphorylating the C-terminal domain (CTD) of RNA Pol II to prevent its recruitment to the promoter and by phosphorylating cyclin H. Cyclin H in complex with CDK7 forms part of the transcription factor complex TFIIH, which in turn phosphorylates the CTD of RNA Pol II, triggering the transition from transcription initiation to mRNA elongation. TFIIH phosphorylates cyclin T as well. The CDK9/cyclin T complex forms part of the positive transcription elongation factor b (pTEFb), which phosphorylates the RNA Pol II CTD, thus promoting the extension of the pre-mRNA.

Transcription is a process that can be influenced at several levels by CDKs, such as with their influence on E2F, [105,118] and the transcription factor FoxM1 during G2 phase by CDK2/cyclin A and CDK1/cyclin B [119,120,121,122]. Also, CDKs are also able to influence the transcription process more directly through regulation of RNA polymerase II (RNA Pol II)-dependent transcription (Figure 2). CDKs can both negatively and positively influence the functionality of RNA Pol II. CDK8 and CDK19 are components of the Mediator complex as part of a 4-subunit subcomplex with cyclin C, Mediator complex subunits MED12 and MED13. This complex acts as an inhibitor of RNA Pol II by phosphorylating its C-terminal domain (CTD), a process which blocks RNA Pol II participation in the pre-initiation complex that drives transcription in eukaryotes [123,124,125]. In contrast to this, there is also CDK-mediated phosphorylation of the RNA Pol II CTD at distinct sites leading to positive regulation of RNA Pol II activity. The pre-initiation complex includes CDK7, its partner cyclin H, and MAT1 (Menage à Trois 1) as a catalytic subunit (named TFIIH) that phosphorylates the CTD of RNA Pol II. This phosphorylation allows for initiation of transcription and elongation to happen [126]. TFIIH, in turn, can also be negatively regulated by CDK8-mediated phosphorylation of cyclin H preventing TFIIH-mediated activatory phosphorylation of the RNA Pol II CTD [67]. CDK9-mediated phosphorylation of the CTD stimulates RNA polymerisation, exerting a positive regulation.

CDK9 promotes phosphorylation of RNA Pol II as a subunit of the positive transcription elongation factor b (pTEFb), where it is found bound to cyclin T1. It also phosphorylates the negative elongation factor NELF, allowing for the extension of the nascent pre-mRNA [127,128]. A mouse study indicates that CDK9 is essential for development and embryonic genome activation. Inhibition of the kinase activity of positive transcription elongation factor b (pTEFb) using the CDK9-specific inhibitor, flavopiridol, caused defects in transcription, abnormal localisation of pTEFb, and developmental arrest in mouse two-cell stage embryos [129]. pTEFb is required for transcription of most genes [130], which explains the drastic consequences observed in this study.

Other CDKs involved in the transcription process are CDK11, 12, and 13. CDK11 has been found to co-immunoprecipitate with the elongation factors ELL2, TFIIF, TFIIS, and FACT, as well as with RNA Pol II itself [31]. Moreover, CDK11 is thought to phosphorylate RNA Pol II on its CTD [131]. CDK12 and CDK13 are also able to phosphorylate RNA Pol II, when in complex with cyclin K [132,133].

Another fundamental process via which CDKs influence transcription is the canonical Wnt pathway. CDK8 kinase activity has been found to be necessary for β-catenin-driven transformation and expression of several β-catenin transcriptional targets [69], while CDK14/cyclin Y complexes are responsible for the phosphorylation of Lrp6, a Wnt coreceptor. This phosphorylation primes Lrp6 for its phosphorylation by the membrane-bound Casein Kinase 1 gamma, leading to the binding of Axin, which is then unable to form part of the destruction complex that ubiquitinylates β-catenin. This chain of events leads to increased TCF/LEF mediated transcription [31].

As such, the examples mentioned above illustrate the diverse roles CDKs play in major cellular processes, and it is likely that more roles of this family will be discovered. It is clear that although their role in the cell cycle has been extensively studied, more research is needed to fully understand CDKs’ integral role in the cell and other central processes in cell biology.

## 5. The Role of CDKs in Pancreatic Cancer

The dysregulation of CDK and cyclin activity in the cell cycle is often found elevated in human tumours and is associated with the unrestrained proliferation of cells, an essential hallmark of cancer [134,135,136]. To maintain these malignant rates at which cells cycle, cancer cells acquire additional mutations which help to circumvent cell cycle checkpoints that usually safeguard genomic integrity and control proliferation [135,136]. A plethora of aberrant CDK behaviour has been described for various tumour entities [44,134,137,138,139]. However, in this review, we will focus on CDK dysregulation described for pancreatic cancer.

Signal transduction cascades activated by proto-oncogene *KRAS*, *CDKN2A* (encoding the tumour suppressors p16INK4a and p14ARF) and *TP53* intersect on the cell cycle, thereby promoting the transition from G0 or G1 phase into S phase [140]. Since pancreatic cancers carry activating mutations in *KRAS*, loss-of-function mutations in *TP53*, and *CDKN2A* in >90%, 50%, and 80% of tumours respectively, controlling CDKs might significantly impact PDAC [3,141,142,143]. So far, histopathological investigations of pancreatic cancer tissue have identified intraepithelial neoplasia (PanIN) lesions which illustrate the multistep genetic progression leading to invasive PDAC. Early stages include the activation of *KRAS* with subsequent inactivation of *TP53* and *CDKN2A/2B* in advanced stages [143].

A crucial checkpoint, controlled by the CDK4/6-cyclin D-Rb pathway, is the transition from G1 to S phase [144]. Consequently, dysregulation of the Rb pathway results in enhanced proliferation [145,146,147,148]. Against the background of a high frequency of *KRAS* mutations, which is the case in PDAC, the CDK4/6 inhibitor encoding function of the *CDKN2A* locus is principally relevant [26]. Despite its oncogenic function, if mutated *KRAS* is expressed in cells with intact cell cycle checkpoints, it triggers a senescent-like state referred to oncogene-induced senescence leading to cell cycle arrest in G1 phase thereby preventing further transformation of the tissue [149,150,151,152]. Oncogene-induced senescence is triggered by either p53 or p16INK4a encoded by *CDKN2A* that inhibits CDK4/CDK6 and prevents the catalytic activity of the CDK/cyclin D complexes [153,154,155,156,157]. CDK4/6-cyclin D-associated hyperphosphorylation of Rb is prevented and consequently S-phase entry through suppression of E2F-mediated transcription [154,158]. In fully developed PDAC, oncogene-induced senescence is bypassed through inactivating of either Rb and/or *TP53* or mutation of p16INK4a [158]. In line with this, germ-line mutations of p16INK4a have been associated with an elevated risk for developing PDAC through uncontrolled CDK4/6-mediated proliferation [149,159]. Besides, recent data underpin a concerted action of mutant KRAS with CDK5 and its activators to intensify malignant progression, migration, and invasion of pancreatic cancer cells [160]. CDK5 holds a unique position within the CDK family, as it becomes activated by the non-cyclin proteins p35 and p39. Although CDK5 shares high homology with CDK2, it has a marginal role in cell cycle control. Most substrates of CDK5 are associated with cell morphology and motility as well as cell–cell communication [161]. Different studies support the relationship between KRAS altered status and CDK inhibition as a potential avenue of treatment in cancer. CDK8 expression was found increased in *KRAS*-mutated pancreatic cancer samples [162], while *KRAS*-mutated PDAC was shown to be sensitive to CDK9-inhibition in patient-derived xenografts [163], and downstream inhibition of KRAS via AKT-inhibition combined with CDK-inhibition via dinaciclib showed dramatic improvements—even complete responses—in patient-derived xenograft models of pancreatic cancer in mice [164]. Of note, CDK4 inhibition was shown to be synthetically lethal with *KRAS* mutations in non-small cell lung cancer (NSCLC), an observation yet to be explored for PDAC [165].

Interestingly, pancreatic acinar cells of adult mice have been found to be resistant to transformation by *KRAS* with or without loss of p16Ink4a/p19Arf or *TP53*, yet they will show PanIN and even PDAC if mice were experimentally induced to develop pancreatitis. If expression of oncogenic *KRAS* was induced after pancreatitis induction, this would only induce PDAC in mice if residual inflammation was still present [166]. In this case, it is thought that the inflammatory response reduces the occurrence of senescence observed in benign lesions. Importantly, it has been found that the CDK4/6 inhibitor palbociclib is able to induce senescence in a breast cancer cell line [167], highlighting CDK inhibition as an interesting avenue of research in senescence induction and its role against oncogenic transformation.

DNA lesions caused by destructive reagents such as chemotherapeutics may trigger checkpoint arrest in the cell cycle and block CDK activity [168,169,170,171,172,173]. DNA damage regularly initiates the p53/p21 pathway resulting in G1 arrest to allow the repair machinery to restore genome stability. There is plenty of evidence in mice and human cells showing the p53-induced regulation of CDK2 by the inhibitor protein p21 [168,173]. Interestingly, it was also shown that apart from that checkpoint element, the binding of CDK2/cyclin E complexes, p21 binds CDK4/cyclin D and suppresses their kinase activities in a p53-dependent manner [174]. Since a vast number of pancreatic tumours carry silencing mutations of *TP53*, this checkpoint is also eliminated.

In case of overexpressed p16, the cellular response is either cell cycle arrest or apoptosis in different pancreatic cancer cell lines [175]. While p16 inhibits CDK4/6 activity leading to growth arrest, p16-induced apoptosis requires activation of p53-dependent cell death pathways [176,177,178]. In line with these findings, earlier results indicated the disruption of CDK4/6 by p16 leading to the rearrangement of the CDK inhibitors p21Cip1 and p27Kip1 [39]. p21/p27 are involved in binding and activity of CDK4/6-cyclin D enzymes, while they are also potent inhibitors of CDK2. p27 loss is frequently associated with tumorigenesis and it has been found that KRAS activation in the pancreas is associated with p27 mislocalization, while the absence of p27 or its ability to bind CDKs triggers the mislocalization of acinar polarity markers related to metaplasia and induces the nuclear expression of transcription factors involved in acinar-to-ductal metaplasia [179]. Also, it was shown that cyclin E overexpression facilitates p16-mediated circumvention of G1 arrest [180,181]. The molecular mechanism of the various fate of pancreatic cancer cells upon CDK4/6 inactivation is associated with the modulation of CDK2 activity [175].

CDK1 monitors M phase entry and exit and binds either cyclin A or cyclin B [134]. In addition to G2/M checkpoint maintenance, the activation of CDK1 is essential to governing mitosis, execution of apoptosis, pluripotency, and genomic stability in human pluripotent stem cells, their proliferation comparable to those of cancer cells [182]. Inactive CDK1, induced for example by cyclin damage, is a condition leading to a shift from M phase and the reconstruction of the G1 phase [183,184]. However, CDK1 activity was identified as essential for tumorigenesis as cell proliferation in transformed cells is mediated by CDK1 [184]. Tumour cells have some proliferation characteristics in common with stem cells; nevertheless, they appear more sensitive to loss of CDK activity [185]. The downregulation of CDK1 in human embryonic stem cells (hESC) and human induced pluripotent stem cells (hiPSC) leads to cell cycle modifications arresting the cells in G2 phase at the expense of G1 and S phase. In both cell types, the loss of the pluripotent stem cell morphology could be detected upon CDK1 downregulation. Knockdown of CDK1 resulted in a significant rise of double-strand breaks [182]. The growth of pancreatic cancer cell lines was reduced when CDC25B, an activator of CDK1, was inhibited resulting in accumulation of phosphorylated CDK1 and G2/M arrest [186].

In addition to their cell cycle-regulating features, a subset of CDKs, most importantly CDK7 and CDK9, have been identified to regulate RNA Pol II-mediated transcription [187,188]. We could recently demonstrate that the upregulation of CDK9 in human pancreatic cancer tissue negatively correlated with patients’ survival. These results became even more significant when the cohort was subdivided by tumour grades revealing a strong correlation between high CDK9 expression levels and reduced overall survival of PDAC patients with well-differentiated tumours (grade 1, grade 2) [139].

## 6. CDK Inhibition in PDAC

Due to their central role in cell cycle control, it is hardly surprising that CDKs and their substrates and regulators are identified as targets of genetic manipulations in various human cancers, which has accelerated the development of small molecule inhibitors against CDKs as an anticancer approach [26,189,190]. The “standard of care” for pancreatic cancer, which means the best treatment known so far, is still disappointing regarding the median overall survival of only six months [17,20,191]. Thus, there is a growing demand for new, more effective treatment possibilities, going in the direction of targeted therapies [26,190]. Of note, unfavourable toxicity profiles and severe adverse effects limited clinical success of potential CDK inhibitor candidates which is thought to be caused by inhibitory activity towards CDK1 which is known to be essential for all living cells [190].

Since the loss of p16INK4a is a standard feature in KRAS-driven PDAC [143], specific pharmacological inhibition of CDK4/6 is of substantial interest and presents a possible target for treatment. So far it has not been possible to design an inhibitor that is completely selective for a specific CDK, due to the lack of three-dimensional structure-displaying models of various CDKs and the high homology within the CDK family.

Currently, the oral CDK4/6-specific compounds palbociclib (PD-0332991) [192], ribociclib (LEE-011) [193], and abemaciclib (LY2835219) [194] are approved for the treatment of breast cancer. They inhibit retinoblastoma (Rb) protein phosphorylation in early G1 phase. Inhibition of Rb phosphorylation, in turn, avoids CDK-mediated G1-S phase transition, thereby arresting the cells in the G1 phase, suppressing DNA synthesis and constraining cancer cell growth. Notably, the approach is likely to succeed in cells exhibiting intact Rb, whereas palbociclib has been demonstrated failing in Rb-negative cancers [192]. Loss-of-function mutations in p16(INK4A) (*CDKN2A*) and therefore in the Rb pathway is a common feature of PDAC, and cause of an early progression in the disease. While this silencing activates CDK4/6, p16(INK4A)-deficient PDAC is nonetheless widely resistant to pharmacological CDK4/6 inhibition [195]. Furthermore, Chou and collaborators showed that inhibition of CDK4 with palbociclib significantly induces apoptosis in pancreatic tumour overexpressing Rb and also enhances the apoptotic effect of chemotherapeutic gemcitabine in patient-derived xenografts in mice. These studies suggest that a combination therapy including CDK4/6 inhibitors could be beneficial in Rb-positive pancreatic tumours and Rb can be used as a tool to select the patients that would benefit the most from this strategy. The approach of CDK4/6 inhibition by palbociclib was also evaluated in combination with IGF1 receptor inhibitors [196] and patient-derived models of pancreatic cancer primary tumour explants. The targeted therapy combinations exhibited robust cytotoxicity towards tumour cells [196]. The CDK4/6 inhibition resulted in potent suppression of tumour growth in the primary tumour explants illustrating the similarities of the biology between primary tumour model and tumour of origin [192]. LY2835219 inhibits CDK4/6 leading to reduced tumour growth in human xenografts, however, not demonstrated up until now for pancreatic cancer. The effect was enhanced in an epithelial tumour xenograft when the inhibitor was administered in combination with gemcitabine [194]. 

Although the underlying mechanism needs to be clarified, metabolic functions organise cell division [197] as the execution of the cell cycle entry is accompanied by an increase in cellular mass and accretion of energetic metabolites necessary for cell division [198]. However, much of the metabolic network is driven by mutated *KRAS* in a concerted manner with tumorigenic proliferation [199,200]. As metabolic characteristics of cancer gradually evolve as a therapeutic target, the CDK4/6 inhibitor-mediated metabolic state might be considered as a therapeutic target [201]. During the cell cycle, CDK4/6 inhibition has different effects but leads to the increase of tumour-associated mitochondrial mass via Rb and reactive oxygen species (ROS). Oxidative and glycolytic metabolic pathways are stimulated upon CDK4/6 inhibition. Apart from that, mTOR signalling is activated as a consequence of CDK4/6 blockade in vivo. These results indicate an active, feed-forward loop involving mTOR pathways for metabolic reprogramming. Consequently, cooperating effects could be shown with mTOR inhibitors resulting in apoptosis induction [201]. The fact that treatment alone with CDK4/6 inhibitors seems not very promising for pancreatic cancer [26], the CDK4/6 inhibition-mediated metabolic state might be additionally targeted [202]. Alternatively, the activity of CDK4/6 inhibitors can be exploited by the combination with mTOR pathway-selective compounds [203]. To overcome the robust apoptotic resistance of pancreatic cancer cells [9], the targeting of the tumour necrosis factor-related, apoptosis-inducing ligand receptors (TRAIL-R1/2) by their ligand TRAIL, in combination with CDK4/6 inhibitors could be used as an anticancer strategy. Indeed, blockage of CDK4/6 sensitises PDAC cells towards TRAIL-induced apoptosis [204]. However, there is additional need for optimisation of these treatment strategies since CDK4/6 inhibition antagonises chemotherapy and radiotherapy as their efficacy mostly relies on the fact that cancer cells undergo fast and uninhibited cell divisions [205,206].

Conversely, there is evidence that release from CDK4/6 inhibition may synchronise cells to go through the cell cycle in a concerted manner and eventually sensitise them to subsequent chemotherapy or may avoid current proliferation or repopulation of cells between treatment cycles [97]. P276-00, a CDK1, CDK4, and CDK9 inhibitor, could sensitise pancreatic cancer cells to gemcitabine-induced apoptosis. The data indicate that the combination might simultaneously target CDKs and Akt/mTOR signalling to inhibit both tumour progress and angiogenesis [207]. Angiogenesis is further influenced by CDK8, which has been found that when overexpressed, it promotes angiogenesis in pancreatic cancer via activation of CDK8/β-catenin/KLF2 signalling, while silencing of CDK8 inhibits angiogenesis in pancreatic cancer in vitro and in nude mice xenograft models [208]. However, the transfer of these hopeful preclinical models to the clinic, where vast genetic tumour heterogeneity and, consequently, dynamics of tumour growth are highly inconsistent between patients, is extremely challenging [209,210].

Dinaciclib (SCH727965) is a small molecule inhibitor with on-target activity in the low nanomolar range against CDK1, CDK2, CDK5, and CDK9. Pre-clinical testing of the substance showed acceptable toxicity and promising efficacy in mouse models and was well tolerated and active in phase I clinical trials [211,212]. Therefore, dinaciclib entered phase III clinical trials with refractory chronic lymphocytic leukaemia (CLL) [213]. In preclinical models for PDAC, monotherapy with dinaciclib inhibited growth, migration, and colony formation of pancreatic cancer cells through the blockage of cell cycle progression and reduction in Rb phosphorylation. Furthermore, this inhibition reduces RalA activity [214]. RalA is an effector of RAS signalling and plays a critical role in tumorigenicity of PDAC, observed for both human and mice [215,216,217]. RalA can be blocked by inactivation of CDK5 probably through effector cascades downstream of oncogenic KRAS. In PDAC xenografts the effects could be verified shown by a significant reduction in tumour growth [214].

Indeed, it has been demonstrated that CDK9 facilitates resistance to apoptosis through the transcriptional control of anti-apoptotic proteins such as Mcl-1 [218]. SNS-032 is a CDK9-selective inhibitor already undergoing clinical testing [219,220,221]. CDK9 was shown to be overexpressed in PDAC tissue and the in vitro response of pancreatic cancer cell lines results in markedly reduced cell viability upon CDK9 inhibition [139]. CDK9 inhibition by SNS-032 might, therefore, pose a promising therapeutic approach for pancreatic cancer. Mechanistically, CDK9 inhibition leads to apoptosis induction by shifting the ratio of anti-apoptotic and pro-apoptotic proteins in line with previous reports describing the inhibition of transcriptional elongation as an antagonist of short-lived anti-apoptotic proteins. As additive effects of CDK inhibition could be achieved in combination with chemotherapeutics, it might be promising to explore this approach as an alternative to conventional treatment strategies [188].

Rohitukine, a plant-derived chromone alkaloid, is the precursor of the promising anticancer clinical candidates flavopiridol (also known as alvocidib, HMR 1275, L86-8275) [222,223,224] and P276-00 [223,225]. However, the antitumor activity, pharmacokinetics, and CDK inhibitory potential of this parental product were not studied in detail for a long time. Eventually, in vitro toxicity could be reported in a variety of cancer cell lines including pancreatic cancer by the inhibition of CDK2 and CDK9 [226]. With the knowledge of the physicochemical properties and targets, the scaffold of rohitukine might be used for the design of further derivates.

## 7. Clinical Trials of CDK-Inhibitors

As discussed above, CDK-inhibition could be a promising strategy for new, advanced therapy protocols to treat pancreatic cancer. There are several ongoing clinical trials with a high number of CDK-inhibitors which we have summarized in Table 2. Unfortunately, pancreatic cancer as an entity is vastly underrepresented in these studies, with only a few results obtained so far.

Flavopiridol (Alvocidib) was one of the first CDK inhibitors, with a wave of publications in the 2000s–2010s. Acting as a pan-CDK-inhibitor (targeting CDK1, 2, 4, 6, 7 and 9) [26], its first results in phase I studies in chronic lymphocytic leukemia (CLL) seemed encouraging, as most of the patients had a reduction in tumour mass; some even had complete responses [27]. Another phase I study, this time in solid tumours, could not confirm this enthusiasm: of 42 subjects, 22 had progression of the disease, 13 had a stable disease, and only seven subjects showed an improvement when flavopiridol was combined with conventional chemotherapy (Oxaliplatin, FOLFOX). Intriguingly, there was one complete response measurable in a case of pancreatic cancer [227]. Another study of this type, with different cytostatics (FOLFIRI; FOL = Folinic acid, F = 5-FU, IRI = Irinotecan), showed similar results [228]. A phase I dose-finding study with docetaxel and flavopiridol in advanced solid tumours showed cases of complete and partial responses towards this regime in pancreatic cancer, but a comparable amount of stable disease [229]. Again, Phase II studies could not confirm these first results. Until now, additional studies have been undertaken in metastatic melanoma, endometrial adenocarcinoma and multiple myeloma, without objective signs of antitumoural activity [190]. Moreover, flavopiridol showed severe adverse effects, and consequently, it was discontinued [240]. Interestingly, flavopiridol in combination with several classical chemotherapeutics, like doxorubicin, docetaxel and FOLFOX (FOL = Folinic acid, F = 5-FU, OX = Oxaliplatin), seems to exacerbate their anti-tumourigenic effect, although this is controversially discussed, as there are also contradictory results on this matter [230,232,258]. One phase II study directed solely towards pancreatic cancer (ClinicalTrials.gov Identifier NCT00331682) showed no benefit from treatment of gemcitabine-refractory, metastatic pancreatic cancer with a combination of docetaxel and flavopiridol [232,233]. Another study (ClinicalTrials.gov Identifier NCT00047307) currently investigates the effects of flavopiridol in combination with radiation, followed by gemcitabine on locally advanced, unresectable pancreatic cancer with no results published so far [231].

Dinaciclib, also acting as a multi-CDK-inhibitor, was first described in 2010 [211]. It showed desirable inhibitory effects towards pancreatic cancer in murine xenograft models [214]. Although clinical studies in humans with pancreatic cancer and dinaciclib are rare, it has been extensively studied in other tumour entities. Dinaciclib is one of the few CDK-inhibitors, next to palbociclib and abemaciclib, which made it past phase II into phase III in CLL [213]. One of the most significant predicted benefits of dinaciclib over flavopiridol is better tolerance over longer timespans, especially in refractory patients [234]. One study showed that with dinaciclib-treatment there is a significant response in CLL patients [234]. Another study demonstrated the efficacy of dinaciclib in relapsed multiple myeloma [237]. In a phase I trial in advanced malignancies dinaciclib was able to stabilise disease while being tolerable over a long time span [212]. In contrast to this, other phase I trials were less favourable. As such, Mitri et al. showed that the combination of dinaciclib with epirubicin could not stop the progression of triple negative breast cancer [235]. Furthermore, this regime came at a price of high toxicity [235]. A phase II study in advanced breast cancer showed capecitabin to be superior over dinaciclib concerning disease-free survival [238]. Dinaciclib showed no antitumoural effects and no superiority over erlotinib in progression-free survival for non-small cell lung cancer in a phase II study [239]. However, most of these studies show good tolerance towards dinaciclib when it is administered as a single drug [212,234,238,239].

Regarding phase III studies, dinaciclib was compared to ofatumumab, an anti-CD-20-antibody [259] in terms of efficacy and safety in relapsed or refractory CLL [213]. This study showed the promising antitumoural activity of dinaciclib in this hematologic neoplasia, with five times better overall response [213]. Currently, there is one already completed phase I study directed towards inoperable pancreatic cancer though there are no results published yet (ClinicalTrials.gov Identifier NCT01783171). In summary, it can be said that while dinaciclib seems like a well tolerable drug, it also seems to show superior anti-tumour activity in non-solid cancers.

SNS-032, a specific inhibitor of CDKs 2, 7, and 9 [219] has only undergone phase I studies until now. It was proven to be tolerable in humans [220]; however, the best clinical response in advanced solid tumours was stable disease in only 15% of cases, whereas the rest of the cases worsened. Again, another phase I study in the hematologic malignancies CLL and multiple myeloma showed better results for SNS-032 treatment [221].

Abemaciclib is a CDK4/6 inhibitor that has made it into phase III clinical studies and is even FDA-approved for certain advanced breast cancers [246]. This drug showed promising results for solid malignancies in several studies, including the extension of progression-free survival [243], even objective response rates in highly advanced breast cancers [242,244]. The JUNIPER study is of particular interest since it investigates the effects of abemaciclib on *KRAS*-mutated non-small cell lung cancer [245]. However, currently, there are no results posted (ClinicalTrials.gov Identifier: NCT02152631). Furthermore, there is an ongoing study targeting the effects of abemaciclib alone or in combination with other agents on previously treated PDAC (ClinicalTrials.gov Identifier: NCT02981342).

The CDK4/6 inhibitor palbociclib is FDA-approved for advanced breast cancers, and most literature refers to this substance in breast cancer and most studies investigate the effects of this substance in breast cancer [250,251,252,253]. Only a few studies addressing pancreatic cancer exist. These studies investigate the effects of combinatory treatments with palbociclib on pancreatic cancer: ClinicalTrials.gov Identifier: NCT02897375 (Cisplatin, Carboplatin), ClinicalTrials.gov Identifier: NCT03454035 (ERK1/2 Inhibitor Ulixertinib), ClinicalTrials.gov Identifier: NCT03065062 (PI3K/mTOR Inhibitor gedatolisib). As of now, no results have been posted yet.

There are ongoing studies with a number of other CDK-inhibitors that include pancreatic cancer: Ribociclib (ClinicalTrials.gov Identifier: NCT02703571; no results posted), the macrolide lactone molecule bryostatin-1 (ClinicalTrials.gov Identifier: NCT00031694; known for activation of protein kinase C, but also inhibition of CDK2 [190,255]; not effective in combination with paclitaxel in advanced pancreatic carcinoma) [256], as well as milciclib was reportedly able to overcome gemcitabine-resistance in a pancreatic cancer patients [257].

In summary, while mostly well tolerable, CDK-inhibitors seem to induce improved responses in hematologic malignancies more than in solid tumours. Reasons for this remain to be explored. Although there are many trials currently ongoing to address the role of CDKs in PDAC, there are still a lot of studies necessary to further evaluate the promise of CDK-inhibitors. For example, the identification of biomarkers, other than Rb, that can predict the response to CDK inhibitor therapies is needed, both to understand in a deeper manner the mechanisms of action of these inhibitors and to determine which patients benefit the most from combinatorial therapies.

CDK/cyclin complexes have been extensively studied in the past decades, yet their roles in the cell cycle and other related processes are not fully understood. It is likely that new functions of CDK/cyclin complexes will be discovered, adding more layers of complexity to the biological consequences of their inhibition, but also shedding more light into the mechanisms of tumorigenesis involved when these proteins expression and activity are altered. As CDK dysregulation is associated with tumorigenesis, these protein remain as an exciting target for therapeutic inhibition. In pancreatic cancer, there is an urgent need for treatment alternatives, and inhibition of CDKs represent a possible approach against this deadly disease.

## Figures and Tables

**Table 1:** Cyclin-Dependent Kinases with corresponding cyclins and their main function.

CDK Subfamily *	Name **	Other Names *	Cyclins	Main Functions	References
CDK1	CDK1	CDC2, CDC28A, cell division cycle 2 homolog A, p34 protein kinase, p34	Cyclin A Cyclin B Cyclin D Cyclin E	Cell cycle—S phase Cell cycle—G2/M phase	[45,46,47,48]
	CDK2	cell division protein kinase 2	Cyclin A Cyclin B Cyclin D Cyclin E	Cell cycle—G1/S transition Cell cycle—S phase	[49,50,51,52]
	CDK3	Cdkn3, Cell division protein kinase 3	Cyclin C Cyclin E	Cell cycle—S phase	[53,54,55]
CDK4	CDK4	cell division protein kinase 4, CRK3, p34/cdk4, PSK-J3	Cyclin D1, D2, D3	Cell cycle—G1 phase Cell cycle—G1/S transition	[56,57,58]
	CDK6	CR2 protein kinase, CRK2, Serine/threonine-protein kinase PLSTIRE	Cyclin D1, D2, D3	Cell cycle—G1 phase Cell cycle—G1/S transition	[56,59,60]
CDK5	CDK5	cell division protein kinase 5, CR6 protein kinase, CRK6, serine/threonine-protein kinase PSSALRE, tau protein kinase II catalytic subunit, TPKII catalytic subunit	p35 *** Cyclin I	Development of the mammalian central nervous system Apoptosis Cell cycle regulation	[61,62,63,64]
CDK7	CDK7	CAK1, CDKN7, STK1, 39 kDa protein kinase, CDK-activating kinase 1, cell division protein kinase 7, CRK4, protein-tyrosine kinase MPK-7, TFIIH basal transcription factor complex kinase subunit	Cyclin H	Transcription regulation	[65,66,67]
CDK8	CDK8	Cell division protein kinase 8, Mediator of RNA polymerase II transcription subunit CDK8	Cyclin C	Transcription regulation Wnt pathway modulation	[68,69,70]
	CDK19	CDC2L6, CDC2-related protein kinase 6, CDK11	Cyclin C	Transcription regulation Cell cycle	[70]
CDK9	CDK9	CDC2L4, cell division protein kinase 9, TAK	Cyclin K Cyclin T1, T2a, T2b	Transcription regulation	[71,72]
CDK10	CDK10	cell division protein kinase 10, PISSLRE	Cyclin M	Transcription regulation	[73]
CRK7	CDK12	CDC2 related protein kinase 7, cell division cycle 2-related protein kinase 7, CRK7, CRKR	Cyclin K Cyclin L	Transcription regulation Splicing	[74,75]
	CDK13	CDC2L, CDC2L5, CDC2-related protein kinase 5, cell division cycle 2-like 5, cell division protein kinase 13	Cyclin K Cyclin L	Transcription regulation Splicing	[74,76]
PITSLRE	CDK11A	CDC2L2, CDC2L3, CDK11-p110, CDK11-p46, CDK11-p58, cell division cycle 2-like 2 (PITSLRE proteins), Cell division cycle 2-like protein kinase 2, Cell division protein kinase 11A, PITSLRE serine/threonine-protein kinase CDC2L2	Cyclin L1, L2	Splicing	[77,78]
	CDK11B	CDC2L1, CDK11-p110, CDK11-p46, CDK11-p58, cell division cycle 2-like protein kinase 1, cell division protein kinase 11, PITSLRE serine/threonine-protein kinase CDC2L1, PITSLRE serine/threonine-protein kinase CDC2L1CDK11-p110	Cyclin L1, L2	Splicing	[77,78]
TAIRE	CDK14	cell division protein kinase 14, PFTAIRE protein kinase 1, PFTAIRE1, PFTK1	Cyclin D Cyclin Y	Cell cycle progression Wnt signaling	[79,80]
	CDK15	ALS2CR7, PFTAIRE protein kinase 2, PFTAIRE2, Cell division protein kinase 15, frizzled family receptor 7		Associated with TRAIL resistance in cancer cells	[81]
	CDK16	cell division protein kinase 16, CRK5, PCTAIRE protein kinase 1, PCTAIRE1	Cyclin Y	Neurite outgrowth regulation Vesicle trafficking Cancer cell proliferation	[82]
	CDK17	PCTAIRE protein kinase 2, PCTAIRE2		Expressed in brain	[83,84]
	CDK18	cell division protein kinase 18, PCTAIRE protein kinase 3, PCTAIRE3	Cyclin A2 Cyclin E	Actin reorganization regulation Genome integrity regulation	[85,86,87]
CCRK	CDK20	CCRK, CDK-related protein kinase PNQLARE, cell division protein kinase 20, Cyclin-dependent protein kinase H, Cyclin-kinase-activating kinase p42		Cell cycle regulation	[88,89]

* according to the classification of Alexander et al. 2017 [90]; ** according to the classification of Malumbres et al. 2009 [37]; *** p35 is not classified in the cyclin family, although p35 and cyclins present a similar three-dimensional conformation that can interact with CDKs [91,92].

**Table 2:** CDK-specific inhibitors currently studied in clinical trials.

Drug	Targeted CDKs	Clinical Trial Phase	Disease	Observations
Flavopiridol	1, 2, 4, 6, 7, 9 [26]	Phase I	Chronic Lymphocytic Leukemia (CLL) [27]	After chemoimmunotherapy
			Advanced solid tumours [227]	In combination with oxaliplatin and fluorouracil/leucovorin
			Advanced solid tumours [228]	In combination with FOLFIRI
			Advanced solid tumours [229]	In combination with docetaxel
			Advanced sarcomas [230]	In combination with doxorubicin
			Pancreatic cancer [231]	In combination with radiation and gemcitabine
		Phase II	Metastatic melanoma, endometrial adenocarcinoma, multiple myeloma [190]	
			Pancreatic cancer [232,233]	In combination with docetaxel
Dinaciclib	1, 2, 5, 9 [211]	Phase I	CLL [234]	
			Advanced malignancies [212]	
			Triple-negative breast cancer [235]	In combination with epirubicin
			Pancreatic cancer [236]	No results published yet
		Phase II	Multiple myeloma [237]	
			Advanced breast cancer [238]	In comparison vs. capecitabine
			Non-small cell lung cancer (NSCLC) [239]	In comparison vs. erlotinib
		Phase III	CLL [213]	In comparison vs. ofatumumab
SNS-032	2, 7, 9 [219]	Phase I	Metastatic refractory solid tumours [220]	
			CLL, multiple myeloma [221]	
Abemaciclib	4, 6 [240]	Phase II	Pancreatic cancer [241]	In combination with gemcitabine, capecitabine
			Advanced breast cancer [242] (MONARCH 1)	
		Phase III	Advanced breast cancer [243] (MONARCH 2)	In combination with fulvestrant
			Advanced breast cancer [244] (MONARCH 3)	In combination with aromatase inhibitor
			NSCLC (KRAS-mutation) [245] (JUNIPER)	In comparison vs. erlotinib
			FDA-approved for advanced breast cancer [246]	
Palbociclib	4, 6 [240]	Phase I	Advanced solid tumours [247,248,249], including pancreatic cancer	In combination with cisplatin, carboplatin, ulixertinib, gedatolisib
		Phase II	Advanced breast cancer [250,251]	In combination with anastrozole, letrozole
		Phase III	Advanced breast cancer [252]	In combination with fulvestrant
			FDA-approved for advanced breast cancer [253]	
Ribociclib	4, 6 [240]	Phase I/II	Advanced solid tumours [254]	
Bryostatin-1	2 [190,255]	Phase II	Advanced pancreatic carcinoma [256]	In combination to paclitaxel
Milciclib	1, 2, 4, 5 [257]	Phase I	Refractory solid tumours [257]	In combination with gemcitabine

## Abbreviations

CAK	CDK-activating kinase
CDK	Cyclin-dependent kinase
CDKN2A	Cyclin-dependent kinase Inhibitor 2A
CLL	Chronic lymphocytic leukemia
CMGC	CDKs, MAPKs, Gsk3β, and CDK-like kinases
CTD	C-Terminal Domain
DYRK	Dual-specificity tyrosine-regulated kinase
ELL2	Elongation Factor for RNA Polymerase II
FACT protein	Facilitates chromatin transcription
FDA	US Food and Drug Administration
FOLFIRINOX	FOL = Folinic acid, L = Leucovorin, F = 5-FU = 5-Fluorouracil, IRIN = Irinotecan, OX = Oxaliplatin
FoxM1	Forkhead box protein M1
GADD45	Growth Arrest and DNA Damage-inducible protein
GSK3β	Glycogen synthase kinase-3 β
hESC	Human embryonic stem cells
hiPSC	Human induced pluripotent stem cells
INK4	Inhibitor of Cyclin-Dependent Kinase 4
MAPK	Mitogen-activated protein kinase
MAT1	Menage á trois 1
Mcl-1	Myeloid leukaemia cell differentiation protein 1
MED12	Mediator complex subunit 12
MED13	Mediator complex subunit 13
MPK7	Mitogen-activated protein kinase 7
mTOR	Mechanistic target of rapamycin
NSCLC	Non-small cell lung cancer
PanIN	Pancreatic intraepithelial neoplasia
PDAC	Pancreatic ductal adenocarcinoma
pTEFb	Positive transcription elongation factor b
RalA	RAS-Like Protein A
Rb	Retinoblastoma protein
RNA Pol II	RNA polymerase II
ROS	Reactive oxygen species
TAK	Tat-associated kinase
TCF/LEF	T-cell factor/lymphoid enhancer factor
TF	Transcription factor
TFIIH	Transcription factor II H
TP53	Tumour protein p53
TPKII	Tau protein kinase II
TRAIL-R1/2	Tumour necrosis factor-related, apoptosis-inducing ligand receptors 1/2
